# Regulation of *Drosophila* Intestinal Stem Cell Proliferation by Enterocyte Mitochondrial Pyruvate Metabolism

**DOI:** 10.1534/g3.119.400633

**Published:** 2019-09-05

**Authors:** Dona R. Wisidagama, Carl S. Thummel

**Affiliations:** Department of Human Genetics, University of Utah School of Medicine, 15 North 2030 East Room 5100, Salt Lake City UT 84112-5330, USA

**Keywords:** mitochondria, intestinal homeostasis, metabolism, stem cells

## Abstract

Multiple signaling pathways in the adult *Drosophila* enterocyte sense cellular damage or stress and signal to intestinal stem cells (ISCs) to undergo proliferation and differentiation, thereby maintaining intestinal homeostasis. Here we show that misregulation of mitochondrial pyruvate metabolism in enterocytes can stimulate ISC proliferation and differentiation. Our studies focus on the Mitochondrial Pyruvate Carrier (MPC), which is an evolutionarily-conserved protein complex that resides in the inner mitochondrial membrane and transports cytoplasmic pyruvate into the mitochondrial matrix. Loss of *MPC* function in enterocytes induces Unpaired cytokine expression, which activates the JAK/STAT pathway in ISCs, promoting their proliferation. Upd3 and JNK signaling are required in enterocytes for ISC proliferation, indicating that this reflects a canonical non-cell autonomous damage response. Disruption of lactate dehydrogenase in enterocytes has no effect on ISC proliferation but it suppresses the proliferative response to a loss of enterocyte MPC function, suggesting that lactate contributes to this pathway. These studies define an important role for cellular pyruvate metabolism in differentiated enterocytes to maintain stem cell proliferation rates.

Like most animals, the intestine of the fruit fly *Drosophila* is a self-renewing tissue in which a population of stem cells generates new differentiated cell types to replace those that are damaged and lost during normal life (Micchelli and Perrimon 2006; Ohlstein and Spradling 2006; Jiang *et al.* 2009; Lemaitre and Miguel-Aliaga 2013). The intestinal stem cells (ISCs) reside in the basal region of the intestine, adjacent to the visceral muscle, and undergo regulated division to maintain tissue homeostasis with complete replacement of the intestinal epithelium every few weeks under normal conditions. Dividing ISCs give rise to a new stem cell through the process of stem cell self-renewal as well as an enteroblast (EB) or secretory enteroendocrine (EE) cell. The EB, in turn, differentiates without cell division into an absorptive enterocyte (EC). The EEs and ECs, with a predominance of ECs, constitute the single cell layer of the intestinal epithelium, with an apical brush border that faces the lumen. This system of regulated stem cell self-renewal and differentiation is conserved through animal evolution, establishing *Drosophila* as an ideal genetic model to define the molecular mechanisms that maintain intestinal physiology and function.

Detailed studies over the past ten years have characterized signaling pathways in the *Drosophila* intestine that maintain proper levels of stem cell proliferation, self-renewal, and differentiation (Bonfini *et al.* 2016; Guo *et al.* 2016; Jiang *et al.* 2016; Miguel-Aliaga *et al.* 2018). These include a stress response pathway in ECs that senses cellular injury or infection and signals to ISCs to promote their proliferation. Acting in part through JNK signaling, this pathway results in the secretion of cytokines of the Unpaired family that activate canonical JAK/STAT signaling in ISCs (Buchon *et al.* 2009a; Jiang *et al.* 2009; Beebe *et al.* 2010; Lin *et al.* 2010). Genetic studies have demonstrated that this response in ISCs is dependent on the Domeless receptor and results in the downstream induction of the canonical JAK/STAT target gene *Socs36E*. The Sox21a transcription factor is also up-regulated by JAK/STAT signaling in ISCs and promotes their proliferation and differentiation (Meng and Biteau 2015; Zhai *et al.* 2015; Zhai *et al.* 2017). These signaling interactions, along with inputs from multiple other pathways, act together to maintain intestinal physiology and function in the adult fly.

Although multiple forms of stress, infection, and damage have been investigated as signals that trigger stem cell proliferation and differentiation, less is known about how changes in cellular metabolism might affect these pathways. Most of these studies have investigated how dietary nutrients can impact intestinal homeostasis. For example, dietary methionine or S-adenosylmethionine synthesis regulates protein synthesis in ISCs and Upd3 cytokine signaling from ECs to support stem cell division in fed animals (Obata *et al.* 2018). Similarly, dietary lipids acting through the Notch signaling pathway regulate EE cell number in the posterior midgut (Obniski *et al.* 2018), and the hexosamine biosynthetic pathway facilitates the proliferative effect of the insulin signaling pathway on ISCs (Mattila *et al.* 2018).

Our studies focus on genetic manipulation of the Mitochondrial Pyruvate Carrier (MPC) as a means of modulating mitochondrial pyruvate metabolism in a cell type-specific manner. The MPC is an evolutionarily-conserved obligate heterodimer of two proteins, MPC1 and MPC2, that resides in the inner mitochondrial membrane and transports pyruvate into the mitochondrial matrix (Bricker *et al.* 2012; Herzig *et al.* 2012). The MPC thus provides a key metabolic step that links cytoplasmic glycolysis with mitochondrial oxidative metabolism. Accordingly, a loss of the MPC results in reduced mitochondrial activity while MPC overexpression leads to increased pyruvate flux into mitochondria and increased oxidative metabolism (Bricker *et al.* 2012; Schell *et al.* 2014). These functions for the MPC place it in a critical position to modulate rates of aerobic glycolysis with downstream effects on cell proliferation. Consistent with this, our past studies have shown that the MPC is both necessary and sufficient in a stem cell autonomous manner to regulate ISC proliferation and maintain intestinal homeostasis in *Drosophila* (Schell *et al.* 2017).

Here we extend our earlier studies of the MPC in the *Drosophila* intestine by addressing roles for cellular pyruvate metabolism in differentiated ECs. We noticed that although a clonal loss of the MPC in ISCs resulted in increased proliferation it did not block their differentiation, leading to the formation of *MPC* mutant enterocytes. This led us to examine the possibility that a loss of the MPC in differentiated intestinal cells might have a non-autonomous effect on wild-type stem cell proliferation. Here we show that cell-specific loss of the *MPC* in ECs acts through the JNK and JAK/STAT signaling pathways to activate ISC proliferation in a non-autonomous manner. These studies establish a role for mitochondrial pyruvate metabolism in ECs to regulate ISC proliferation rates.

## Materials and Methods

### Fly strains

*Drosophila* stocks were maintained on standard food containing 3% sucrose, 6% glucose, 8% yeast, and 1% agar in a 25° incubator. For *dMPC1* genetic studies, control flies were homozygous for a precise excision of the *P(XP)CG14290[d00809] P*-element insertion and mutant flies were transheterozygous for the two deletion alleles, *dMPC1^1^* and *dMPC1^2^*, as described (Bricker *et al.* 2012). Transgenic lines are as follows: *UAS-dMPC1-RNAi* (Bricker *et al.* 2012), *UAS-PDH-E1-RNAi* (NIG 7010R-3), *UAS-PDH-E2-RNAi* (NIG 5261R-3), *UAS-LDH-RNAi* (Bloomington 33640), *UAS-Bsk^DN^* (Bloomington 6409), *UAS-upd3-RNAi* (Bloomington 28575), *upd3**.1-lacZ* (Jiang *et al.* 2011), and *10XSTAT92E-DGFP* (Bach *et al.* 2007). The *UAS-LDH-RNAi* transgenic line has been used previously for functional studies of this enzyme (Slaninova *et al.* 2016; Charlton-Perkins *et al.* 2017). *Tub-GAL80^ts^* was used to restrict RNAi to the adult stage in all experiments. The *10XSTAT92E-DGFP* and *w*; *myo1A-GAL4/CyO*; *Tub-GAL80^ts^*, *UAS-GFP/TM6B* stocks were a gift from B. Edgar.

### Histology and immunostaining

Intestines were dissected in 1xPBS and fixed with 4% formaldehyde (Polysciences Inc, EM grade) overnight at 4°. Tissues were washed four times with 0.1% Triton, 1xPBS (PBST) and incubated with appropriate primary antibodies at 4° overnight. Antibodies used: mouse anti-beta galactosidase (DSHB 40-1a) diluted 1:100 in PBST, chicken anti-GFP (Abcam ab13970) diluted 1:10,000, and rabbit anti-phospho-histone H3 (pHH3) diluted 1:1000 (Millipore 06-570). The tissues were then washed and incubated for 3-4 hr with the following secondary antibodies: donkey anti-mouse Cy3-conjugated antibodies (Jackson 715-165-157), donkey anti-rabbit Cy3-conjugated antibodies (Jackson 715-165-152), or goat anti-chicken Alexa-488 antibodies (Abcam ab150169). Samples were mounted using Vectashield (Vector, USA) with DAPI. Images were acquired using an Olympus FV1000 confocal microscope and assembled into Z stack projections for the figures.

### RNA-seq analysis

Adult *myo1A > mCherry-RNAi* animals (controls) and *myo1A > dMPC1-RNAi* animals, both with *Tub-GAL80^ts^* to restrict RNAi to the adult stage, were aged for 7 days. Four biological replicates of 15 intestines per genotype were dissected in 1x cold PBS and immediately transferred to Trizol on ice. RNA was extracted as described (Zymo Research RNA extraction kit R2051) and RNA quality was analyzed using a Agilent RNA ScreenTape assay. Total RNA from each biological replicate was subjected to Illumina HighSeq2000 50-cycle single-read sequencing. Standard replicate RNA-seq analysis was performed using USeq and DESeq analysis packages with alignment to the *D. melanogaster* dm3 genome assembly. Transcripts displaying a log2-fold change ≥0.6 in mRNA abundance and FDR ≤1% were considered as differentially expressed genes. RNA quality control, library preparation, sequencing, and data analysis were performed at the University of Utah High Throughput Genomics and Bioinformatics Core Facilities.

### GC-MS analysis

Control and *dMPC1* mutant animals were raised until 7 days of adulthood. Twenty females per sample were snap-frozen in liquid nitrogen and prepared for analysis by gas chromatography/mass spectrometry (GC/MS) as described (Tennessen *et al.* 2014). Data are presented from four independent experiments, each consisting 20 animals per condition. Sample preparation and GC/MS analysis were performed by the Metabolomics Core Research Facility at the University of Utah School of Medicine.

### N-acetyl cysteine feeding

Five day old adults were transferred to vials supplemented with 0.1% NAC (Sigma A7250) and shifted to 29°. Animals were maintained on this NAC supplemented diet for 7 days after which the intestines were dissected and subjected to antibody stains to detect pHH3.

### Statistical analysis

GraphPad PRISM 6 software was used to plot data and perform statistical analysis. Pairwise comparison p-values were calculated using a two-tailed Student’s *t*-test. Multiple comparison P values were calculated using one-way ANOVA with Sidak’s multiple test correction. Error bars depict the mean ± SEM.

### Data availability

A full list of all RNAi lines screened can be found above with the corresponding stock numbers. All reagents are also cited with regard to catalog number and source. RNA-seq data from this study can be accessed at NCBI GEO (accession number: GSE136211). Supplemental material available at FigShare: https://doi.org/10.25387/g3.9621263.

## Results and Discussion

### Mitochondrial pyruvate metabolism in enterocytes regulates stem cell proliferation

**Figure 1:**
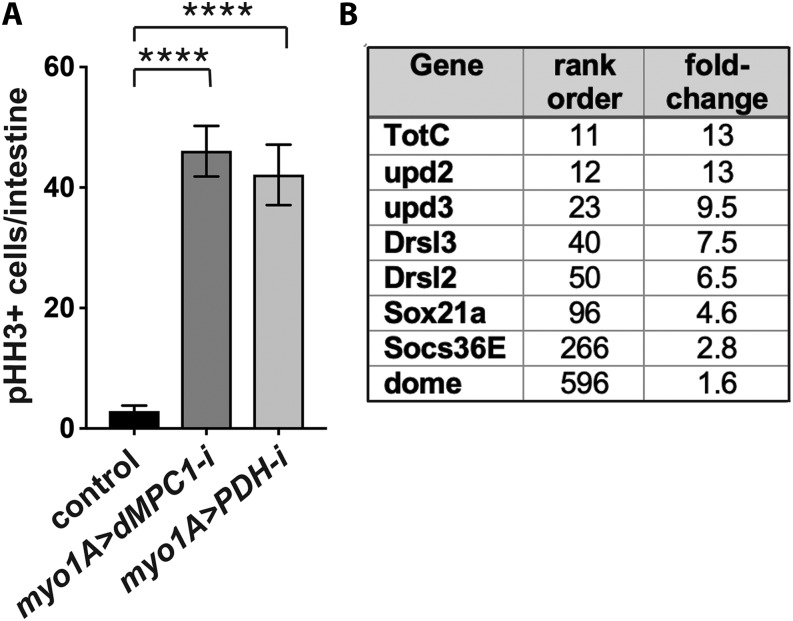
Reduced mitochondrial pyruvate metabolism in enterocytes increases stem cell proliferation in a non-autonomous manner. (A) The EC-specific *myo1A-GAL4* driver was used to target RNAi for *mCherry* (control), *dMPC1*, or *PDH-E1* in the presence of *Tub-GAL80^ts^*. The number of cells staining positive for phosphorylated histone H3 (pHH3+ cells) was quantified per intestine after shifting 4-5 day old animals to 29°C for seven days. Data are plotted as the mean ± SEM, n ≥ 20 animals for each condition, ****P ≤ 0.0001. (B) Genes that encode key components in the JAK/STAT signaling pathway increase their expression in *myo1A > dMPC1-RNAi* intestines. Data from Supplementary Table 1 is depicted for genes involved in JAK/STAT signaling in the intestine, showing their rank order in the RNA-seq dataset and fold-change increase in expression relative to the control.

Previously we demonstrated that loss of MPC function in ISCs enhances their proliferation in a cell autonomous manner (Schell *et al.* 2017). During these studies we noticed that GFP-marked *dMPC1* mutant MARCM clones included mutant stem cells that differentiated into mature ECs or EEs. This observation raised the possibility that mutant differentiated cells might signal to adjacent wild-type ISCs, inducing their proliferation in a non-cell autonomous manner. To test this hypothesis, we targeted *dMPC1* RNAi specifically to ECs using the *myo1A-GAL4* driver and measured ISC proliferation by staining dissected intestines for phosphorylated histone H3 (pHH3) (Figure 1A, Supplementary Figure 1A). RNAi was temporally restricted to the adult stage by using *Tub-GAL80^ts^* (McGuire *et al.* 2004). Interestingly, this experiment revealed a major increase in ISC proliferation, more than twofold higher than we had seen with ISC-specific *Dl** > dMPC1* RNAi under similar conditions (Figure 1A, Supplementary Figure 1A) (Schell *et al.* 2017). This observation supports the conclusion that a loss of mitochondrial pyruvate metabolism in differentiated ECs can impact ISC proliferation rates.

To confirm that this response is due to changes in EC pyruvate metabolism, we conducted a similar experiment using *myo1A-GAL4* to target RNAi for a gene encoding part of the pyruvate dehydrogenase (PDH) complex. PDH acts downstream from the MPC in the mitochondrial matrix where it catalyzes the conversion of pyruvate into acetyl-CoA, which is the initial metabolite in the tricarboxylic acid (TCA) cycle. Quantification of pHH3-staining cells in *myo1A > PDH* RNAi intestines revealed a major increase in ISC proliferation, similar to that seen with *dMPC1* RNAi (Figure 1A). Taken together, these results indicate that disruption of pyruvate metabolism in ECs is sufficient to induce ISC proliferation in a non-cell autonomous manner.

To gain insights into the molecular mechanisms by which ECs might be signaling to ISCs, we conducted genome-wide RNA sequencing analysis using dissected intestines from *dMPC1* mutants and *myo1A > dMPC1-RNAi* animals (Supplementary Table 1). Interestingly, the *myo1A > dMPC1-RNAi* dataset included increased expression of multiple canonical markers of the JAK/STAT pathway (Figure 1B). This included the inflammatory cytokine genes *upd2* and *upd3* among the top most affected genes, as well as the inflammatory-responsive Drosomycin genes *Drsl2* and *Drsl3*, the gene encoding the Domeless cytokine receptor, and the canonical JAK/STAT target genes *Sox21a* and *Socs36E* (Figure 1B). This response is consistent with the well-established signaling pathway by which stressed or damaged ECs signal to ISCs to promote their proliferation and differentiation (Buchon *et al.* 2009a; Buchon *et al.* 2009b; Jiang *et al.* 2009; Beebe *et al.* 2010; Lin *et al.* 2010).

### Enterocytes require Upd3 and JNK signaling to indirectly control stem cell proliferation

**Figure 2:**
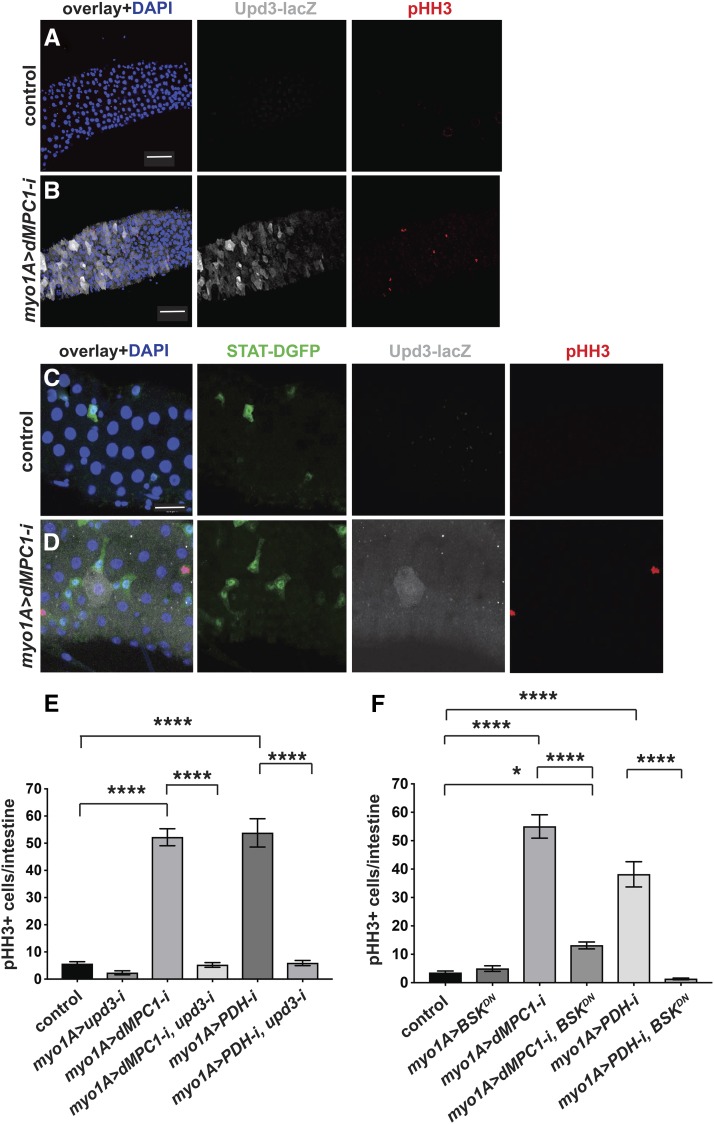
*MPC* loss of function in enterocytes requires UPD3 and JNK to indirectly control stem cell proliferation. The *myo1A-GAL4* driver was used to target RNAi for *mCherry* (control) (A,C) or *dMPC1* (B,D) in the presence of *Tub-GAL80^ts^*. The *upd3**.1-lacZ* reporter (gray, A-D) and *10xSTAT92E-DGFP* reporter (green, C,D) were used to detect Upd3 expression and JAK/STAT activation, respectively, by immunohistochemistry. Proliferating ISCs are marked by staining for pHH3+ cells (red) and nuclei are stained with DAPI (blue). Panels 2A,B and 2C,D are from independent experiments and depict the R4 region of the intestine. Scale bars represent 50 µm (A,B) and 20 µm (C,D). (E) The *myo1A-GAL4* driver was used to target RNAi for *mCherry* (control), *dMPC1*, *PDH-E1*, or *upd3*, either alone or in combination as shown, in the presence of *Tub-GAL80^ts^*. The number of cells staining positive for phosphorylated histone H3 (pHH3+ cells) was quantified per intestine after shifting 4-5 day old animals to 29°C for seven days. Data are plotted as the mean ± SEM, n ≥ 20 animals for each condition. (F) The *myo1A-GAL4* driver was used to target *mCherry* RNAi (control), *dMPC1* RNAi, *PDH-E2* RNAi, or *Bsk^DN^* expression, either alone or in combination as shown, in the presence of *Tub-GAL80^ts^*. The number of cells staining positive for phosphorylated histone H3 (pHH3+ cells) was quantified per intestine after shifting 4-5 day old animals to 29°C for seven days. Data are plotted as the mean ± SEM, n ≥ 20 animals for each condition. ****P ≤ 0.001, *P ≤ 0.05.

If ECs initiate cytokine signaling in response to a loss of the MPC then we would expect to see increased *upd3* expression in these cells accompanied by elevated JAK/STAT signaling and proliferation in ISCs. To test this possibility, we used *myo1A-GAL4* to target *dMPC1* RNAi in ECs in the presence of the *upd3**.1-lacZ* and *10XSTAT92E-DGFP* reporters (Bach *et al.* 2007; Jiang *et al.* 2011). As expected, *myo1A > dMPC1* RNAi resulted in increased ISC proliferation relative to controls as detected by pHH3 antibody staining (Figure 2A-D, red nuclei). In addition, ECs displayed varying levels of elevated *upd3**.1-lacZ* expression. Moreover, we could detect cells adjacent to these ECs that display high levels of *10XSTAT92E-DGFP* expression. Although we did not determine the identity of these GFP-expressing cells, their small size and triangular shape suggest that they represent ISCs, pre-ECs, or EBs (Figure 2D). Taken together, these observations are consistent with canonical cytokine signaling from ECs to ISCs, activating the JAK/STAT pathway and promoting ISC proliferation.

Genetic studies have demonstrated that *upd3* is the main cytokine that controls JAK/STAT signaling in the intestine (Osman *et al.* 2012). We therefore set out to determine if *upd3* is required in ECs for the effect on ISC proliferation. As expected, *myo1A > dMPC1* RNAi resulted in a dramatic increase in proliferating cells in the intestine (Figure 2E) and this response was unaffected by including nonspecific *mCherry* RNAi in the genetic background (Supplementary Figure 1B). EC-specific *myo1A > **upd3* RNAi alone had no effect on proliferation rates relative to the control (Figure 2E). However, the elevated proliferation seen with *myo1A > dMPC1* RNAi was eliminated by including *upd3* RNAi in the genetic background (Figure 2E). A similar effect was seen when *myo1A-GAL4* was used to drive *PDH* RNAi and *upd3* RNAi in ECs (Figure 2E). Taken together these observations support the model that a loss of mitochondrial pyruvate metabolism in the ECs acts through Upd3 cytokine signaling to activate stem cell proliferation.

The JNK pathway is activated by cellular damage and stress in ECs and required for Upd3 cytokine expression (Buchon *et al.* 2009a; Jiang *et al.* 2009). We therefore asked if the non-cell autonomous control of stem cell proliferation caused by changes in EC pyruvate metabolism is dependent on JNK signaling. For this purpose, we used *myo1A-GAL4* to drive the expression of a dominant-negative form of JNK using the *UAS-Bsk^DN^* construct in either the presence or absence of *UAS-dMPC1-RNAi* or *UAS-PDH-RNAi*. EC-specific expression of *Bsk^DN^* had no effect on the basal rate of ISC proliferation (Figure 2F). In contrast, combining this expression with either *dMPC1* RNAi or *PDH* RNAi resulted in a major reduction in ISC proliferation (Figure 2F). These observations indicate that JNK signaling is required in ECs for the non-cell autonomous effect of EC mitochondrial pyruvate metabolism on ISC proliferation.

### dMPC1 mutants have elevated levels of lactate

Although a number of studies have addressed how stress signaling pathways in ECs can promote ISC proliferation, much less is known about how metabolic changes might impact this response. One possibility is that the mitochondrial dysfunction resulting from reduced levels of pyruvate oxidation might lead to an increase in oxidative stress. However, feeding the dietary antioxidant N-acetylcysteine (NAC) to animals with EC-specific *dMPC1* RNAi had no effect on the rate of ISC proliferation (Supplementary Figure 1C). A similar treatment of *dSdhaf3* mutants in our lab was sufficient to rescue the oxidative stress caused by a loss of Succinate Dehydrogenase activity (Na *et al.* 2014). Although further studies are needed to definitively conclude that oxidative stress is not contributing to the non-autonomous effect of EC metabolism on ISC proliferation, this result provides support for this possibility.

**Figure 3:**
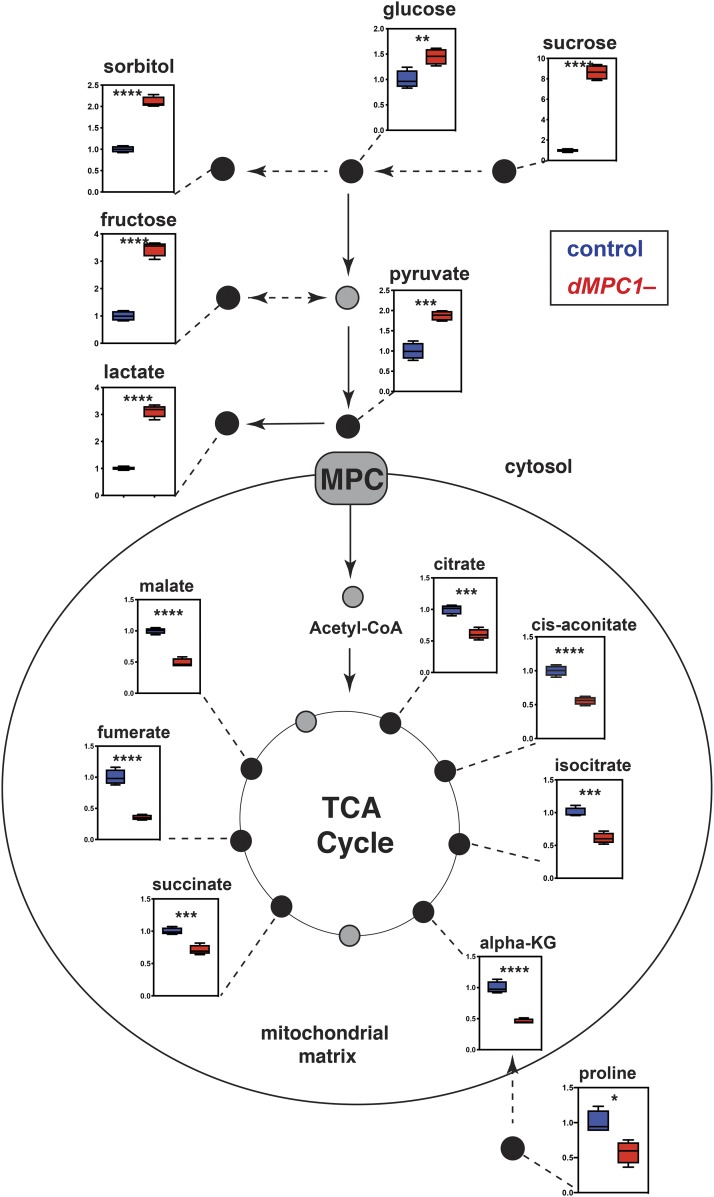
*dMPC1* mutants have increased glycolytic intermediates and reduced TCA cycle intermediates. GC/MS metabolomic profiling of controls (blue bars) and *dMPC1* mutants (red bars) on a diet containing 9% sugar and 8% yeast. Data are graphically represented as a box plot, with the box representing the lower and upper quartiles, the horizontal line representing the median, and the bars representing the minimum and maximum data points. Mutant values are normalized to the control and fold changes in metabolite levels are displayed. n = 4 independent experimental collections. ****P ≤ 0.0001, ***P ≤ 0.001, **P ≤ 0.01 *P ≤ 0.05.

An alternative model is that changes in the level of one or more metabolite in *MPC* mutant ECs might provide a signal that is sensed by the stress response pathway. Although we attempted to perform untargeted GC/MS metabolomic analysis on dissected *Drosophila* intestines we were unable to generate reproducible data using this approach. Accordingly, we used GC/MS to profile basic metabolites in extracts from whole control animals and *dMPC1* mutants. We used mature adult females maintained on a diet containing 8% yeast 3% sucrose and 6% glucose for seven days in order to reproduce the experimental conditions used for the functional studies in this paper. As expected, the loss of *MPC* function leads to a backup in glycolytic intermediates, including elevated levels of glucose, sorbitol, pyruvate, and lactate (Figure 3). This is accompanied by reduced levels of TCA cycle intermediates along with reduced proline, which is a major amino acid for anaplerotic input into the TCA cycle in insects (Teulier *et al.* 2016). These results are consistent with our previous observation that *dMPC1* mutants are hyperglycemic and display shifts in the levels of metabolic intermediates that are consistent with reduced mitochondrial pyruvate import (Bricker *et al.* 2012; McCommis *et al.* 2016).

### LDH is required in enterocytes for non-cell autonomous effects on stem cell proliferation

**Figure 4:**
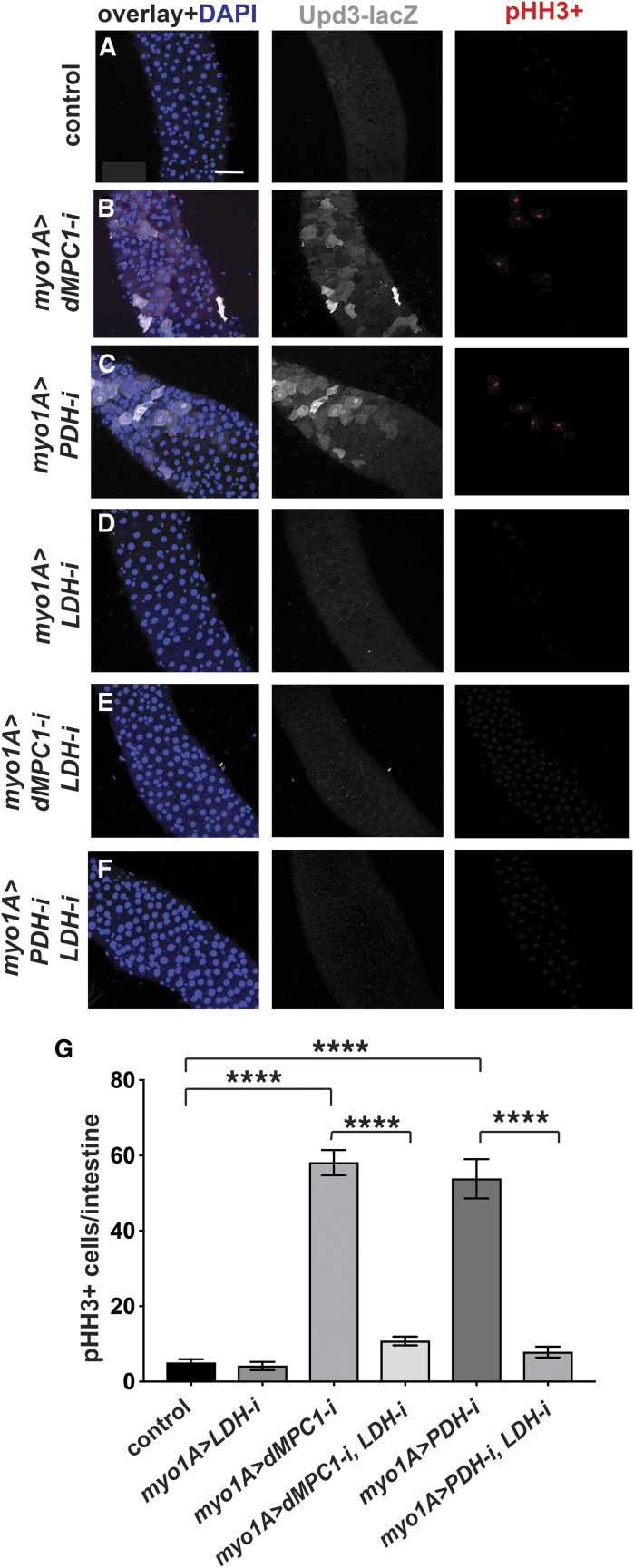
LDH is required in enterocytes for the non-autonomous effect of *MPC* loss on stem cell proliferation. (A-F) The *myo1A-GAL4* driver was used to target RNAi for *mCherry* (A), *dMPC1* (B,E), *PDH-E1* (C,F), and *LDH* (D-F) in the presence of *Tub-GAL80^ts^*. The *upd3**.1-lacZ* reporter (gray) was used to detect Upd3 expression, proliferating ISCs are marked by staining for pHH3+ cells (red), and nuclei are stained with DAPI (blue). Images depict the R4 region of the intestine. Scale bar represents 50 µm. (G) The *myo1A-GAL4* driver was used to target RNAi for *mCherry* (control), *LDH*, *dMPC1*, or *PDH-E1* in the presence of *Tub-GAL80^ts^*. The number of cells staining positive for phosphorylated histone H3 (pHH3+ cells) was quantified per intestine after shifting 4-5 day old animals to 29°C for seven days. Data are plotted as the mean ± SEM, n ≥ 20 animals for each condition. ****P ≤ 0.0001.

We noted from our metabolomic analysis that lactate is increased ∼threefold in *dMPC1* mutants compared to controls (Figure 3). Clearly, whole animal metabolomic analysis might not reflect metabolic changes that take place in specific intestinal cell types. Nonetheless, this provides a starting point to address how a shift in cellular metabolite levels due to a loss of the MPC might lead to a stress response in ECs. In addition, lactate production by LDH plays an important role in mediating the effects of hexosamine biosynthesis on ISC proliferation in the *Drosophila* intestine (Mattila *et al.* 2018). Moreover, changes in LDH activity could affect NAD levels and thus indirectly impact cellular redox state, which has been shown to regulate intestinal JNK signaling and ISC proliferation (Hochmuth *et al.* 2011; Wang *et al.* 2014). We thus set out to determine if LDH might be required for the increased expression of Upd3 in ECs lacking the MPC. We used *myo1A-GAL4* to direct *LDH* RNAi in ECs in the presence of the *upd3**.1-lacZ* reporter. Under these conditions we saw no detectable Upd3 expression, similar to controls (Figure 4A,D). Adding *dMPC1* RNAi to this genetic background, however, revealed that the enhanced expression of Upd3 seen upon a loss of the MPC in ECs is suppressed by including *LDH RNAi* (Figure 4B,E). A similar effect was observed when *myo1A-GAL4* was used to drive *PDH* RNAi in the presence or absence of *LDH* RNAi (Figure 4C,F). Importantly, we also saw a non-cell autonomous effect on ISC proliferation as measured by staining for pHH3 (Figure 4A-F). Quantitation of this effect revealed that the increased ISC proliferation seen upon *dMPC1* or *PDH* RNAi in ECs is effectively suppressed by including *LDH* RNAi in these genetic backgrounds (Figure 4G). Taken together, these observations suggest that lactate production is required in ECs for the elevated expression of the Upd3 cytokine in response to reduced mitochondrial pyruvate metabolism.

### Reduced EC pyruvate metabolism indirectly regulates ISC proliferation

**Figure 5:**
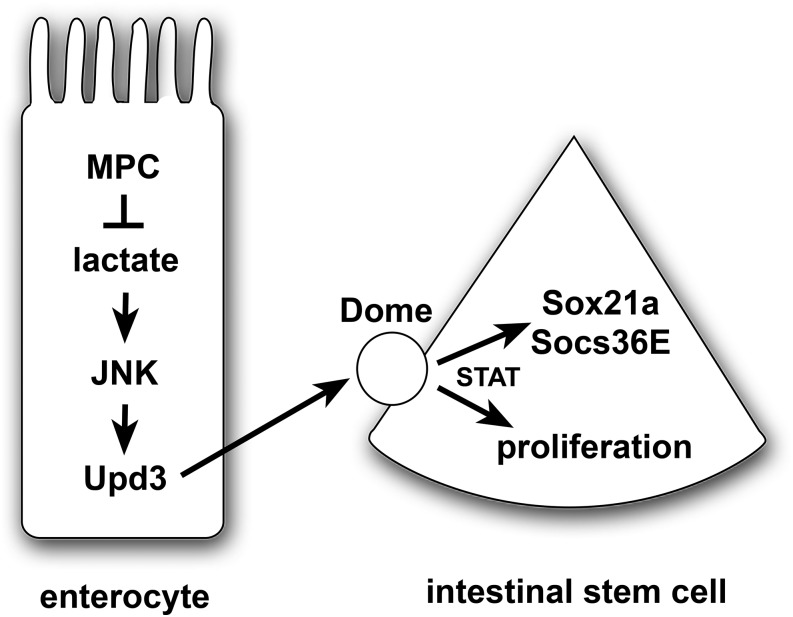
A model for the non-autonomous regulation of ISC proliferation by enterocyte pyruvate metabolism. A loss of the MPC in enterocytes results in increased lactate production by LDH, which activates the JNK stress signaling pathway and Upd3 expression. This secreted cytokine acts through the Dome receptor in ISCs to promote JAK/STAT signaling, resulting in up-regulation of Sox21a and Socs36E as well as stem cell proliferation. Note that this is a speculative model that is intended to summarize the conclusions from this study in the context of the well-established JNK/STAT stress response pathway in the intestine.

In conclusion, our studies demonstrate that a disruption in mitochondrial pyruvate metabolism in ECs activates JAK/STAT signaling resulting in a non-cell autonomous effect on ISC proliferation. Our work supports the model that elevated lactate levels generated by LDH are an important stress signal in ECs that lack MPC function (Figure 5). We cannot, however, rule out other roles for LDH in these pathways, such as its contributions to NAD production or 2-hydroxyglutarate synthesis (Hochmuth *et al.* 2011; Wang *et al.* 2014; Li *et al.* 2017). This stress acts through the canonical JNK pathway to result in Upd3 cytokine expression that, in turn, can signal to ISCs. This signaling activates the JAK/STAT pathway which promotes stem cell proliferation, resulting in the production of new differentiated intestinal cells that can replace those cells that have mitochondrial dysfunction. These functional interactions parallel those of other studies that have demonstrated a critical role for JNK and JAK/STAT signaling in maintaining intestinal homeostasis under conditions of tissue stress, infection, or damage (Jiang and Edgar 2011; Miguel-Aliaga *et al.* 2018). Our results are also consistent with prior reports that mitochondrial function is important for intestinal homeostasis in response to stress and aging in *Drosophila* (Koehler *et al.* 2017; Deng *et al.* 2018). This work expands these studies to include a specific role for pyruvate metabolism in ECs and provides a foundation for future efforts aimed at understanding how changes in cellular physiology can modulate intestinal homeostasis and function.

## References

[bib1] Bach E. A., Ekas L. A., Ayala-Camargo A., Flaherty M. S., Lee H., 2007 GFP reporters detect the activation of the Drosophila JAK/STAT pathway in vivo. Gene Expr. Patterns 7: 323–331. 10.1016/j.modgep.2006.08.003 17008134

[bib2] Beebe K., Lee W. C., and Micchelli C. A., 2010 JAK/STAT signaling coordinates stem cell proliferation and multilineage differentiation in the Drosophila intestinal stem cell lineage. Dev. Biol. 338: 28–37. 10.1016/j.ydbio.2009.10.045 19896937

[bib3] Bonfini A., Liu X., and Buchon N., 2016 From pathogens to microbiota: How Drosophila intestinal stem cells react to gut microbes. Dev. Comp. Immunol. 64: 22–38. 10.1016/j.dci.2016.02.008 26855015

[bib4] Bricker D. K., Taylor E. B., Schell J. C., Orsak T., Boutron A., 2012 A mitochondrial pyruvate carrier required for pyruvate uptake in yeast, Drosophila, and humans. Science 337: 96–100. 10.1126/science.1218099 22628558 PMC3690818

[bib5] Buchon N., Broderick N. A., Chakrabarti S., and Lemaitre B., 2009 a Invasive and indigenous microbiota impact intestinal stem cell activity through multiple pathways in Drosophila. Genes Dev. 23: 2333–2344. 10.1101/gad.1827009 19797770 PMC2758745

[bib6] Buchon N., Broderick N. A., Poidevin M., Pradervand S., and Lemaitre B., 2009 b Drosophila intestinal response to bacterial infection: activation of host defense and stem cell proliferation. Cell Host Microbe 5: 200–211. 10.1016/j.chom.2009.01.003 19218090

[bib7] Charlton-Perkins M. A., Sendler E. D., Buschbeck E. K., and Cook T. A., 2017 Multifunctional glial support by Semper cells in the Drosophila retina. PLoS Genet. 13: e1006782 10.1371/journal.pgen.1006782 28562601 PMC5470715

[bib8] Deng H., Takashima S., Paul M., Guo M., and Hartenstein V., 2018 Mitochondrial dynamics regulates Drosophila intestinal stem cell differentiation. Cell Death Discov. 4: 17 10.1038/s41420-018-0083-0 PMC6056485 30062062

[bib9] Guo Z., Lucchetta E., Rafel N., and Ohlstein B., 2016 Maintenance of the adult Drosophila intestine: all roads lead to homeostasis. Curr. Opin. Genet. Dev. 40: 81–86. 10.1016/j.gde.2016.06.009 27392294 PMC5135564

[bib10] Herzig S., Raemy E., Montessuit S., Veuthey J. L., Zamboni N., 2012 Identification and functional expression of the mitochondrial pyruvate carrier. Science 337: 93–96. 10.1126/science.1218530 22628554

[bib11] Hochmuth C. E., Biteau B., Bohmann D., and Jasper H., 2011 Redox regulation by Keap1 and Nrf2 controls intestinal stem cell proliferation in Drosophila. Cell Stem Cell 8: 188–199. 10.1016/j.stem.2010.12.006 21295275 PMC3035938

[bib12] Jiang H., and Edgar B. A., 2011 Intestinal stem cells in the adult Drosophila midgut. Exp. Cell Res. 317: 2780–2788. 10.1016/j.yexcr.2011.07.020 21856297 PMC6141237

[bib13] Jiang H., Grenley M. O., Bravo M. J., Blumhagen R. Z., and Edgar B. A., 2011 EGFR/Ras/MAPK signaling mediates adult midgut epithelial homeostasis and regeneration in Drosophila. Cell Stem Cell 8: 84–95. 10.1016/j.stem.2010.11.026 21167805 PMC3021119

[bib14] Jiang H., Patel P. H., Kohlmaier A., Grenley M. O., McEwen D. G., 2009 Cytokine/Jak/Stat signaling mediates regeneration and homeostasis in the Drosophila midgut. Cell 137: 1343–1355. 10.1016/j.cell.2009.05.014 19563763 PMC2753793

[bib15] Jiang H., Tian A., and Jiang J., 2016 Intestinal stem cell response to injury: lessons from Drosophila. Cell. Mol. Life Sci. 73: 3337–3349. 10.1007/s00018-016-2235-9 27137186 PMC4998060

[bib16] Koehler C. L., Perkins G. A., Ellisman M. H., and Jones D. L., 2017 Pink1 and Parkin regulate Drosophila intestinal stem cell proliferation during stress and aging. J. Cell Biol. 216: 2315–2327. 10.1083/jcb.201610036 28663346 PMC5551703

[bib17] Lemaitre B., and Miguel-Aliaga I., 2013 The digestive tract of Drosophila melanogaster. Annu. Rev. Genet. 47: 377–404. 10.1146/annurev-genet-111212-133343 24016187

[bib18] Li H., Chawla G., Hurlburt A. J., Sterrett M. C., Zaslaver O., 2017 Drosophila larvae synthesize the putative oncometabolite L-2-hydroxyglutarate during normal developmental growth. Proc. Natl. Acad. Sci. USA 114: 1353–1358. 10.1073/pnas.1614102114 28115720 PMC5307464

[bib19] Lin G., Xu N., and Xi R., 2010 Paracrine unpaired signaling through the JAK/STAT pathway controls self-renewal and lineage differentiation of drosophila intestinal stem cells. J. Mol. Cell Biol. 2: 37–49. 10.1093/jmcb/mjp028 19797317

[bib20] Mattila, J., K. Kokki, V. Hietakangas and M. Boutros, 2018 Stem Cell Intrinsic Hexosamine Metabolism Regulates Intestinal Adaptation to Nutrient Content. Dev Cell 47**:** 112–121 e113. 10.1016/j.devcel.2018.08.011 PMC6179903 30220570

[bib21] McCommis K. S., Hodges W. T., Bricker D. K., Wisidagama D. R., Compan V., 2016 An ancestral role for the mitochondrial pyruvate carrier in glucose-stimulated insulin secretion. Mol. Metab. 5: 602–614. 10.1016/j.molmet.2016.06.016 27656398 PMC5021712

[bib22] McGuire S. E., Mao Z., and Davis R. L., 2004 Spatiotemporal gene expression targeting with the TARGET and gene-switch systems in Drosophila. Sci. STKE 2004: pl6 10.1126/stke.2202004pl6 14970377

[bib23] Meng F. W., and Biteau B., 2015 A Sox Transcription Factor Is a Critical Regulator of Adult Stem Cell Proliferation in the Drosophila Intestine. Cell Reports 13: 906–914. 10.1016/j.celrep.2015.09.061 26565904

[bib24] Micchelli C. A., and Perrimon N., 2006 Evidence that stem cells reside in the adult Drosophila midgut epithelium. Nature 439: 475–479. 10.1038/nature04371 16340959

[bib25] Miguel-Aliaga I., Jasper H., and Lemaitre B., 2018 Anatomy and Physiology of the Digestive Tract of Drosophila melanogaster. Genetics 210: 357–396. 10.1534/genetics.118.300224 30287514 PMC6216580

[bib26] Na U., Yu W., Cox J., Bricker D. K., Brockmann K., 2014 The LYR factors SDHAF1 and SDHAF3 mediate maturation of the iron-sulfur subunit of succinate dehydrogenase. Cell Metab. 20: 253–266. 10.1016/j.cmet.2014.05.014 24954417 PMC4126850

[bib27] Obata, F., K. Tsuda-Sakurai, T. Yamazaki, R. Nishio, K. Nishimura *et al.*, 2018 Nutritional Control of Stem Cell Division through S-Adenosylmethionine in Drosophila Intestine. Dev Cell 44**:** 741–751 e743. 10.1016/j.devcel.2018.02.017 29587144

[bib28] Obniski R., Sieber M. and Spradling A. C., 2018 Dietary Lipids Modulate Notch Signaling and Influence Adult Intestinal Development and Metabolism in Drosophila. Dev. Cell 47: 98–111.e5. 10.1016/j.devcel.2018.08.013 30220569 PMC6894183

[bib29] Ohlstein B., and Spradling A., 2006 The adult Drosophila posterior midgut is maintained by pluripotent stem cells. Nature 439: 470–474. 10.1038/nature04333 16340960

[bib30] Osman D., Buchon N., Chakrabarti S., Huang Y. T., Su W. C., 2012 Autocrine and paracrine unpaired signaling regulate intestinal stem cell maintenance and division. J. Cell Sci. 125: 5944–5949. 10.1242/jcs.113100 23038775

[bib31] Schell J. C., Olson K. A., Jiang L., Hawkins A. J., Van Vranken J. G., 2014 A role for the mitochondrial pyruvate carrier as a repressor of the Warburg effect and colon cancer cell growth. Mol. Cell 56: 400–413. 10.1016/j.molcel.2014.09.026 25458841 PMC4268416

[bib32] Schell J. C., Wisidagama D. R., Bensard C., Zhao H., Wei P., 2017 Control of intestinal stem cell function and proliferation by mitochondrial pyruvate metabolism. Nat. Cell Biol. 19: 1027–1036. 10.1038/ncb3593 28812582 PMC6137334

[bib33] Slaninova V., Krafcikova M., Perez-Gomez R., Steffal P., Trantirek L., 2016 Notch stimulates growth by direct regulation of genes involved in the control of glycolysis and the tricarboxylic acid cycle. Open Biol. 6: 150155 10.1098/rsob.150155 26887408 PMC4772804

[bib34] Tennessen J. M., Barry W. E., Cox J., and Thummel C. S., 2014 Methods for studying metabolism in Drosophila. Methods 68: 105–115. 10.1016/j.ymeth.2014.02.034 24631891 PMC4048761

[bib35] Teulier L., Weber J. M., Crevier J., and Darveau C. A., 2016 Proline as a fuel for insect flight: enhancing carbohydrate oxidation in hymenopterans. Proc. Biol. Sci. 283: 20160333 10.1098/rspb.2016.0333 27412285 PMC4947884

[bib36] Wang L., Zeng X., Ryoo H. D., and Jasper H., 2014 Integration of UPRER and oxidative stress signaling in the control of intestinal stem cell proliferation. PLoS Genet. 10: e1004568 10.1371/journal.pgen.1004568 25166757 PMC4148219

[bib37] Zhai Z., Boquete J. P., and Lemaitre B., 2017 A genetic framework controlling the differentiation of intestinal stem cells during regeneration in Drosophila. PLoS Genet. 13: e1006854 10.1371/journal.pgen.1006854 28662029 PMC5510897

[bib38] Zhai Z., Kondo S., Ha N., Boquete J. P., Brunner M., 2015 Accumulation of differentiating intestinal stem cell progenies drives tumorigenesis. Nat. Commun. 6: 10219 10.1038/ncomms10219 26690827 PMC4703904

